# Atrophy of the lateral geniculate nucleus in human glaucoma detected by magnetic resonance imaging

**DOI:** 10.1136/bjo.2008.138172

**Published:** 2008-08-12

**Authors:** N Gupta, G Greenberg, L Noël de Tilly, B Gray, M Polemidiotis, Y H Yücel

**Affiliations:** 1 Ophthalmology & Vision Sciences, Faculty of Medicine, University of Toronto, Toronto, Canada; 2 Laboratory Medicine & Pathobiology, Faculty of Medicine, University of Toronto, Toronto, Canada; 3 Glaucoma and Nerve Protection Unit, St Michael’s Hospital, University of Toronto, Toronto, Canada; 4 Keenan Research Center at the Li Ka Shing Knowledge Institute of St Michael’s Hospital, University of Toronto, Toronto, Canada; 5 Division of Neuroradiology, Department of Diagnostic Imaging, St Michael’s Hospital, University of Toronto, Toronto, Canada; 6 Ophthalmic Pathology Laboratory, University of Toronto, Toronto, Canada

## Abstract

**Aim:**

To determine in vivo whether the lateral geniculate nucleus (LGN) undergoes atrophy in patients with glaucoma and vision loss compared with normal subjects.

**Methods:**

Following institutional St Michael’s Hospital Research Ethics Board approval, a prospective and masked neuroimaging study was conducted on glaucoma patients with visual-field defects affecting both eyes (n = 10) and age-matched controls (n = 8). Following informed consent, all subjects underwent 1.5-Tesla MRI. Coronal proton density magnetic resonance images of both LGNs were obtained, and LGN height measurements were measured by consensus by three neuroradiologists masked to the diagnosis. Glaucoma and control groups were compared using the t test.

**Results:**

Both LGNs were identified and visualised by 1.5-Tesla MRI for every subject. Compared with controls, the mean LGN heights in glaucoma were decreased in right (4.09 (0.89) mm vs 4.74 (0.54) mm, p>0.05) and left LGNs (3.98 (0.57) mm vs 4.83 (0.95) mm; p = 0.033). The combined right and left LGN height in glaucoma was significantly decreased compared with controls (8.07 (1.06) mm vs 9.56 (0.86) mm; p = 0.005).

**Conclusion:**

In vivo MRI evidence of LGN degeneration in human glaucoma is consistent with ex vivo primate and human neuropathological studies. LGN atrophy may be a relevant biomarker of visual system injury and/or progression in some glaucoma patients.

Glaucoma is a leading cause of world blindness, and its pathological hallmark is retinal ganglion cell (RGC) loss. Current therapies are directed at lowering intraocular pressure, and this helps to slow disease progression.^1^ Although the RGC cell body lies within the eye, the large part of its axon lies outside the eye, forming the optic nerve, chiasm and optic tract. Ninety per cent of the RGCs terminate in the lateral geniculate nucleus (LGN), the major relay station between the retina and visual cortex.^2^ In experimental monkey glaucoma with optic nerve fibre loss, the LGN undergoes degenerative changes, including overall LGN shrinkage and reduced neuron size and numbers.^3^^–^6 These findings provide evidence of trans-synaptic degeneration in glaucoma, and may be relevant to understanding disease spread in select patients.^7^

Neuroimaging studies of the LGN in the context of vision loss are rare.^8^^–^11 Anatomical challenges include its small size and maximal diameter of 4–6 mm^12^ and proximity to other adjacent grey-matter thalamic nuclei. Technical challenges include optimisation of MR imaging parameters to consistently identify and discriminate the LGN from surrounding white- and grey-matter structures.^13^ ^14^

In a post-mortem human glaucoma case with bilateral visual-field loss, reduced LGN and neuron size were observed by histomorphometry and ex vivo MRI, compared with age-matched controls.^15^ It is not known whether LGN size in glaucoma is reduced in vivo compared with normal subjects. In glaucoma patients with similar bilateral visual-field loss, we prospectively assessed the LGN size in vivo by 1.5-Tesla MRI compared with age-matched controls.

## PATIENTS AND METHODS

### Subjects

Following institutional research ethics board approval, informed consent was obtained. Glaucoma subjects (n = 10) were recruited prospectively from the Glaucoma and Nerve Protection Unit at St Michael’s Hospital, University of Toronto. Inclusion criteria required a diagnosis of primary open-angle glaucoma, with evidence of glaucomatous optic neuropathy and documented visual-field loss involving both eyes. Exclusion criteria included a history of non-glaucomatous ocular disease, or neurological disorder.

Ten age-matched control subjects were recruited mainly from hospital personnel and, after informed consent, underwent complete eye examination and visual-field testing. Inclusion criteria included normal eye exam and visual fields. Subjects with a history of ocular or neurological disease were excluded. MRIs of eight control subjects were used in this study, as one subject did not attend the scheduled session, and another was discovered to have a history of previously treated ocular hypertension.

There was no statistically significant difference in mean age between the glaucoma and control groups (63.1 (SD 7.7) years vs 58.6 (10.0) years; p>0.05) (table 1). In the glaucoma group, Humphrey 24-2 visual field MD ranged from −5.06 dB to −20.43 dB, and there was no statistically significant difference in mean deviation between OD and OS (−12.63 (4.18) dB vs −15.76 (4.30) dB; p>0.05). In the glaucoma group, the vertical cup/disc ratio ranged from 0.5 to 0.9 and there was no statistically significant difference in cup/disc ratio between OD and OS (0.79 (0.12) vs 0.77 (0.15); p>0.05). Compared with the control group, MDs and cup/disc ratio (table 1) were significantly different in the glaucoma group for both eyes (p<0.05).

**Table 1:** Details of normal and glaucoma subjects

	Age	Sex	Mean deviation (dB)		Cup/disc ratio	
			OD	OS	OD	OS
Control						
C1	71	M	0.45	0.05	0.5	0.3
C2	68	F	3.13	0.53	0.3	0.3
C3	52	M	1.44	1.17	0.3	0.3
C4	46	F	1.9	1.99	0.2	0.2
C5	54	F	−0.44	−1.69	0.3	0.3
C6	65	M	1.05	3.07	0.7	0.7
C7	66	F	1.17	0.93	0.5	0.5
C8	47	F	−0.96	−2	0.7	0.7
Mean	58.6		0.97	0.51	0.44	0.41
SD	10.0		1.30	1.72	0.19	0.20
Glaucoma						
G1	66	M	−11.07	−10.93	0.8	0.6
G2	66	M	−17.23	−15.97	0.8	0.8
G3	52	F	−5.06	−17.13	0.8	0.8
G4	65	F	−11.83	−8	0.7	0.6
G5	60	F	−20.43	−22.1	0.8	0.8
G6	58	F	−10.45	−18.88	0.9	0.9
G7	76	F	−11.17	−17.3	0.8	0.9
G8	52	M	−14.52	−14.44	0.5	0.5
G9	65	F	−10.88	−20.02	0.9	0.9
G10	71	F	−13.69	−12.82	0.9	0.9
Mean	63.1		−12.63	−15.76	0.79	0.77
SD	7.7		4.18	4.30	0.12	0.15
t Test (p value)	NS		0.005	0.005	0.0002	0.0005

OD, right eye; OS, left eye.

### Magnetic resonance imaging

**Figure 1:**
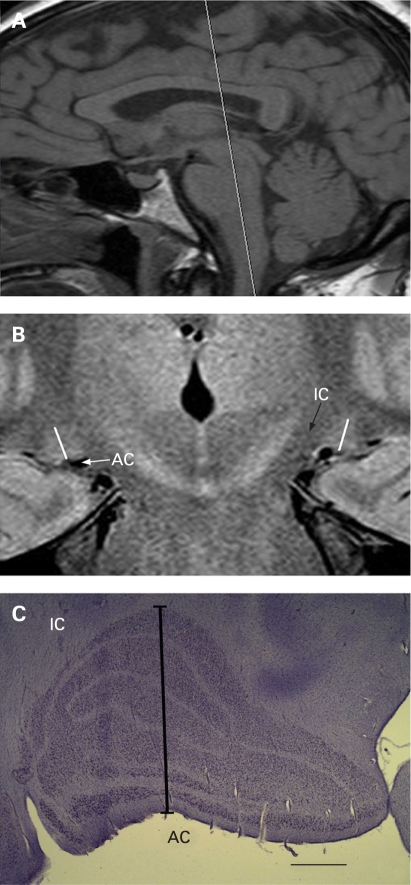
(A) Sagittal T1 image showing the slice orientation for coronal scans parallel to the brainstem reference line. (B) Coronal 2 mm proton density weighted LGN image showing orientation used for height measurement (white oblique lines). (C) Nissl stained coronal LGN section showing orientation used for height measurement (black line). Surrounding anatomical structures are indicated as IC (posterior limb of internal capsule) and AC (ambient cistern). The calibration bar indicates 1 mm.

MRI studies were performed on a 1.5-Tesla MRI scanner (Philips Intera release 1.11, Eindhoven, Netherlands) using an eight-channel volume head coil. Preliminary experiments were performed using both inversion recovery and proton density sequences, and images with proton density measurement were found to be consistently better at visualising the LGN.^13^ ^14^ Multiple sequences were applied: sagittal T1 (TR/TE 747/14, TSE factor of 5, slice thickness 4 mm gap 10 mm, 256×256 matrix, default slice order acquisition, NEX of 3); axial FLAIR (TR/TE 11000/140, slice thickness 6 mm gap 1 mm, 256×256 matrix, TSE Factor of 27, default slice order acquisition, NEX of 1); coronal proton density (TR/TE 3000/12, slice thickness 2 mm gapless, 256×256 matrix, TSE factor of 7, default slice order acquisition, NEX of 5); DWI (TR/TE 3000/75, slice thickness 6 mm, default slice order acquisition, NEX of 4). All sequences were fast spin echoes or EPI for the Diffusion series. Sagittal T1 and axial FLAIR images of the brain were obtained for optimal spatial orientation and to rule out any incidental abnormality along the visual pathways. LGN images were acquired in the coronal plane orthogonal to the long axis of the brainstem reference line (fig 1A).

All scans were of approximately 30 min duration for each patient, and performed by the same technician, on two separate days. Thirty-six LGNs of 18 patients were readily detected on proton density MR images, giving a bright signal intensity surrounded by low-signal-intensity white-matter tracts, with anatomical boundaries that included the lateral recess of the ambient cistern, the posterior limb of the internal capsule and the optic radiations. In all subjects each LGN was visible on at least two consecutive images. For each of the 36 LGNs, the section with the largest cross-sectional area was selected independently by each of the three neuroradiologists with 100% agreement. Image analysis was performed by three neuroradiologists who were masked to the diagnosis and who were able to access coronal images of the LGN only, without optic nerves and optic chiasm. MR image data were analysed using Magicweb software (Siemens AG, Medical Solutions, Health Services. Version: 17.09.2003, Munich, Germany). LGN height was obtained by drawing a perpendicular line from the apex of the convexity to the base of the nucleus by consensus of three neuroradiologists masked to the diagnosis in one session (fig 1B). To reflect input from both eyes with no significant difference in cup/disc ratio or mean deviation between OD and OS in glaucoma, combined right and left LGN heights were calculated for each subject. Glaucoma and control groups were compared using the t test.

### Neurohistological measurement of LGN height

After research ethics board approval, post-mortem brain specimens (n = 4, mean age 74.4 (9.9) years, ranging from 62 to 85 years) were obtained. The left cerebral hemispheres were used for neuropathological examination, and neurological diseases were ruled out. Brain specimens containing the right LGN were cryoprotected, frozen and serially sectioned (50 μm thick) with a sliding microtome in the coronal plane perpendicular to the optic tract.^3^ Every 15th section was stained with Nissl. Using the section with the largest cross-sectional area, LGN height was measured by drawing a line from the apex of the convexity to the base of the nucleus in a perpendicular fashion with morphometry software (Neurolucida software, MicroBrightField, Williston, Vermont) and bright-field microscope (SM51, Olympus, Tokyo) (fig 1C). LGN height measurements from MRI scans were also determined by drawing a line from the apex of the convexity to the base of the nucleus in a perpendicular fashion.

## RESULTS

**Figure 2:**
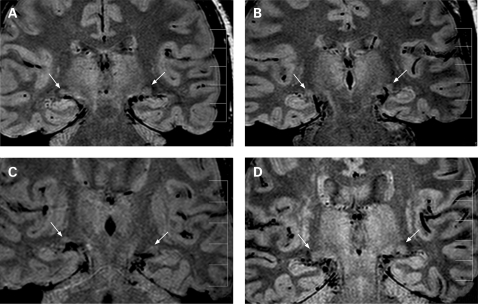
Representative coronal proton density weighted lateral geniculate nucleus (LGN) images in control #C3 (A) and glaucoma subjects with lowest (B, #G1) average (C, #G6) and highest (D, #G2) LGN heights. The arrows indicate right and left LGNs. Calibration bar indicates 50 mm with 10 mm intervals.

In all control and glaucoma subjects, both LGNs were identified, and examples are given in fig 2.

**Figure 3:**
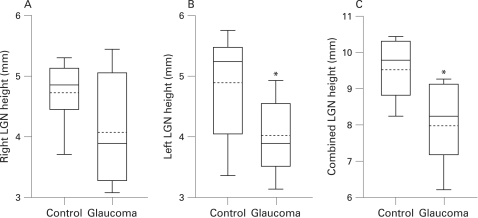
Box plots of right, left and combined lateral geniculate nuclei (LGN) in A, B and C, respectively. The box extends from the 25th percentile to the 75th percentile, with horizontal solid and dotted lines at the median and mean, respectively. The bars indicate the highest and lowest values.

In control subjects, right and left LGN heights by 1.5-Tesla MRI ranged from 3.71 mm to 5.31 mm and from 3.36 mm to 5.74 mm, respectively (table 2). The mean right and left LGN heights were 4.74 (0.54) mm and 4.83 (0.95) mm, respectively, and this difference was not statistically different (p>0.05) (fig 3).

**Table 2:** Right, left and combined lateral geniculate nucleus (LGN) heights for glaucoma and control patients

	Right LGN height (mm)	Left LGN height (mm)		Combined LGN height (mm)	
Control					
C1	4.89		3.36*		8.25*
C2	5.23		5.07		10.3
C3	3.71*		5.74		9.45
C4	4.83		5.49		10.32
C5	5.03		5.42		10.45
C6	4.21*		4.67		8.88
C7	4.68		5.46		10.14
C8	5.31		3.4*		8.71*
Mean	4.74		4.83		9.56
SD	0.54		0.95		0.86
95% lower confidence limit	4.29		4.03		8.84
Glaucoma					
G1	3.35*		3.7*		7.05*
G2	4.32		4.92		9.24
G3	3.18*		4.69		7.87*
G4	3.08*		3.13*		6.21*
G5	3.46*		3.82*		7.28*
G6	3.47*		3.97*		7.44*
G7	4.49		4.14		8.63*
G8	5.24		3.79*		9.03
G9	5.45		3.28*		8.73*
G10	4.87		4.39		9.26
Mean	4.09		3.98		8.07
SD	0.89		0.57		1.06
t Test (p value)	0.09		0.033		0.005

*LGN height is below the 95% lower confidence limit of the control group.

Neurohistological measurements of the LGN in four different normal post-mortem human brain specimens showed a mean maximum LGN height of 4.9 (0.83) mm. This value is similar to in vivo MRI measures of the LGN for normal subjects.

In glaucoma, right and left LGN heights by 1.5-Tesla MRI ranged from 3.08 mm to 5.45 mm and from 3.13 mm to 4.92 mm, respectively (table 2). The mean right and left LGN heights were 4.09 (0.89) mm and 3.98 (0.57) mm, respectively, and this difference was not statistically different (p>0.05).

The mean right LGN height in glaucoma was decreased compared with that observed in controls, but this difference did not reach statistical significance (4.09 (0.89) mm vs 4.74 (0.54) mm, p>0.05) (fig 3). Fifty per cent of glaucoma subjects were below the lower 95% confidence limit of the control group (table 2).

The mean left LGN height in glaucoma was decreased compared with that observed in controls, and this difference was statistically significant (3.98 (0.57) mm vs 4.83 (0.95) mm; p = 0.033) (fig 3). Sixty per cent of glaucoma subjects were below the lower 95% confidence limit of the control group (table 2).

The combined LGN height (right+left) was calculated to account for input from each glaucomatous eye to both LGNs. The combined LGN height in normal and glaucoma subjects ranged from 8.25 mm to 10.45 mm and from 6.21 mm to 9.26 mm, respectively. Compared with the glaucoma group, the mean combined LGN height was decreased, and this difference was statistically significant (8.07 (1.06) mm vs 9.56 (0.86) mm; p = 0.005) (fig 3). Seventy per cent of glaucoma subjects were below the lower 95% confidence limit of the control group (table 2).

## DISCUSSION

Compared with other elements along the visual axis, neuroimaging data relating to the intrinsic structure of the LGN are scarce, mainly due to its location and small size. Previous studies to discriminate the LGN using 3 mm axial thickness could recognise the LGN on only one axial image, with obscuration of the medial border by the medial geniculate nucleus.^13^ ^14^ By using the coronal plane,^16^ in combination with 2 mm thick slices, the LGN was visible on at least two and sometimes three scans in our study. The improved visualisation described in this article may be relevant to detailed assessment of LGN pathology in various diseases.^17^^–^19 The LGN height measured by MRI in normal subjects was similar to histomorphometric measurements of the LGN obtained from normal post-mortem brain specimens. This suggests that LGN height, readily measured by MRI, might be a tool to assess LGN size in health and disease.

There is evidence that the LGN undergoes degenerative changes in experimental primate^3^^–^6 and human^15^ glaucoma. At the macroscopic level, there is obvious atrophy of the LGN.^3^ ^15^ Histomorphometric measurements indicate neuron shrinkage and death affecting magno- and parvocellular LGN neurons.^3^^–^6 Thus, findings in this in vivo neuroimaging study of glaucoma patients with moderate visual-field loss demonstrating LGN atrophy are in keeping with histological studies showing reduced size and neural degeneration in experimental and human glaucoma.

Seven out of 10 glaucoma patients and two of eight control patients showed a combined LGN height below the lower 95% confidence limit of the control group. This suggests that at the present time, this measurement cannot be used for diagnosis of glaucoma or to assess neural degeneration of the LGN in glaucoma for an individual patient in cross-sectional studies. Larger studies are needed to determine LGN variation in normal populations with gender and age considerations, and to further understand the contribution of LGN pathology to vision loss in glaucoma. Since, in this study, all glaucoma patients had moderate to advanced vision loss, it is not possible to correlate LGN heights with disease severity. Longitudinal studies are required to determine whether LGN height reduces with disease progression. This structural MRI study in glaucoma may also be relevant toward the application of functional MRI studies of the LGN in normal and disease states that affect visual pathways.^14^ ^20^ Reduced LGN size in glaucoma using readily available 1.5-Tesla MRI provides in vivo evidence of LGN atrophy in glaucoma patients with moderate visual-field loss.

Neurodegenerative diseases such as Alzheimer disease are associated with progressive cerebral atrophy, and this can be assessed by MRI.^21^ In vivo MRI linear measures including the maximum height of the hippocampus have been used in Alzheimer disease assessment.^22^ ^23^ Neuroimaging research in Alzheimer disease also involves longitudinal MRI studies to track disease progression and exploits MRI to better classify and stage disease.^21^ Further neuroimaging studies of LGN atrophy in glaucoma may provide new insights into sites of injury and progressive disease, with LGN atrophy as a potential biomarker in some cases of moderate to severe glaucoma.
